# Supplementary material for: Genetically Predicted Type 2 Diabetes Mellitus Liability, Glycated Hemoglobin and Cardiovascular Diseases: A Wide-Angled Mendelian Randomization Study
Source: Genes (Basel). 2021 Oct 19;12(10):1644. doi: 10.3390/genes12101644 (PMC8536164; doi:10.3390/genes12101644)
Supplement: Supplementary file 1 [file genes-12-01644-s001.zip › genes-1419563-supplementary.pdf]

SUPPLEMENTARY MATERIALS

(a)

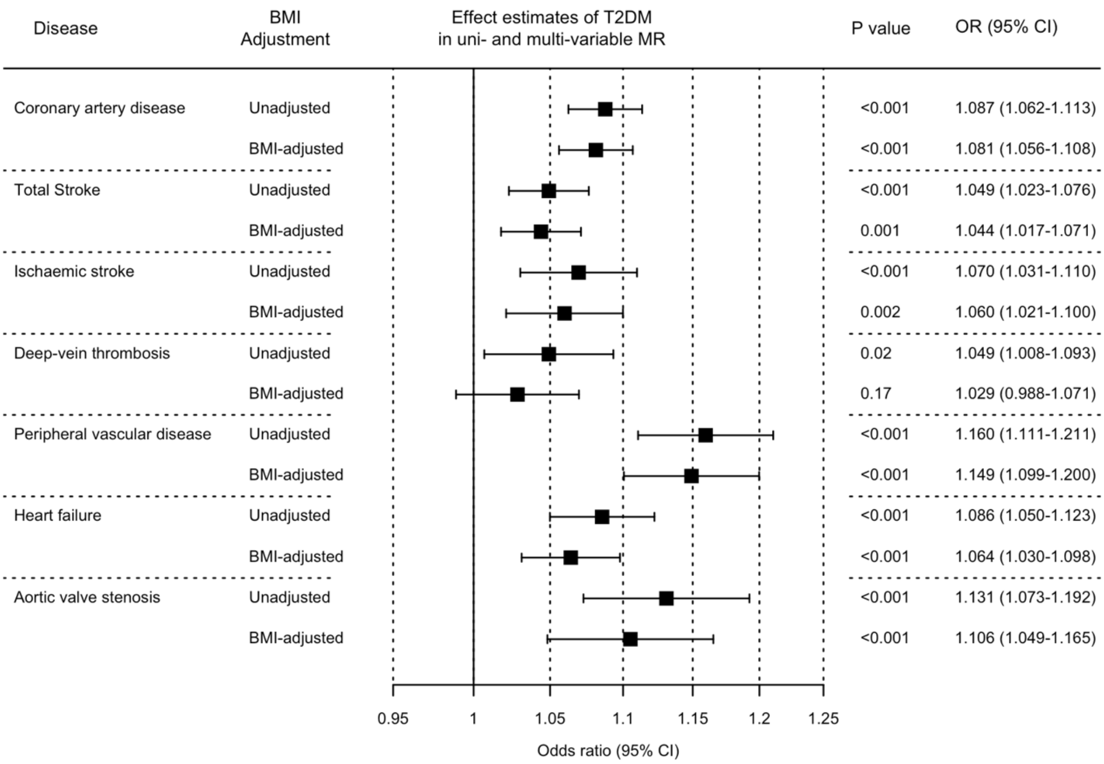

(b)

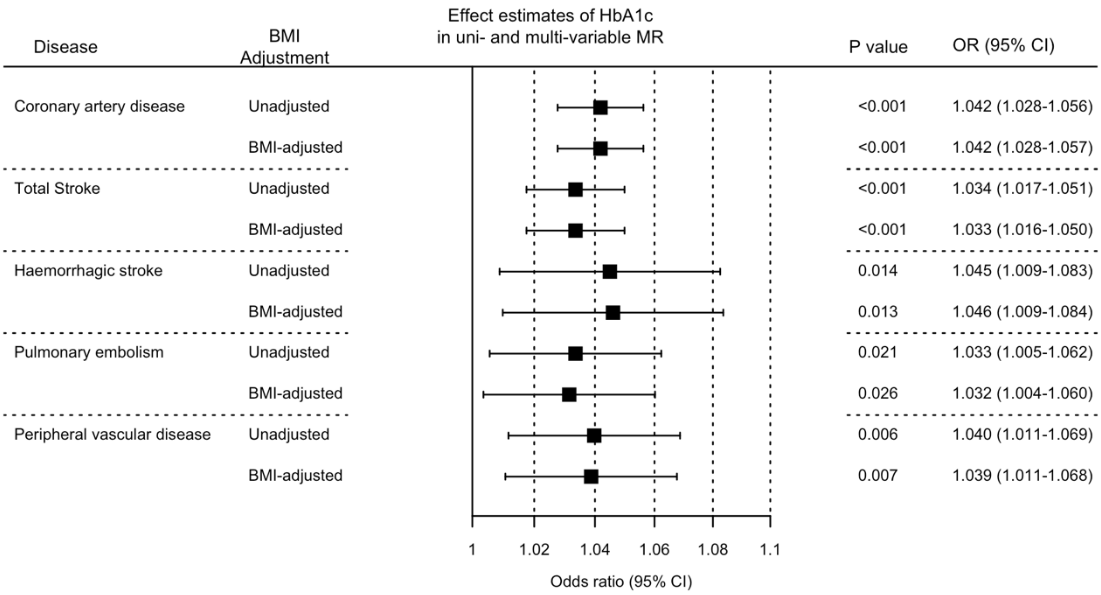

Supplementary Figure S1. Comparison between Mendelian randomization estimates of type 2 diabetes (panel a) mellitus and HbA1c (panel b) on CVD outcomes before and after the adjustment for body mass index (BMI).

Outcomes were selected for being associated with T2DM or HbA1c with a significant or suggestively significant p-value in the primary MR analyses. Multivariable MR analyses were performed to estimate the impacts of T2DM and HbA1c with adjustment for BMI. The corresponding unadjusted estimates were from univariable MR analyses using the same sets of genetic variant

Table S1. Summary of case numbers and definitions of included cardiovascular outcomes based on classification codes in UK Biobank.

Disease name	Number of cases*	Health conditions from electronic health records			Self-reported health conditions			
		ICD-9 Diagnosis	ICD-10 Diagnosis	Operative procedures (OPCS4)	Self-reported non-cancer illness code (20002)	Self-reported operation code (20004)	Code of vascular and bronchiolar conditions diagnosed by doctor (6152)	Code of vascular and heart problems diagnosed by doctor (6150)
Coronary artery disease	29278	410.X, 411.X, 412.X, 414.0, 414.8, 414.9	I21.X, I22.X, I23.X, I24.X, I25.1, I25.2, I25.5, I25.6, I25.8, I25.9	K40.X, K41.X, K42.X, K43.X, K44.X, K45.X, K46.X, K49.X, K50.1, K50.2, K50.4, K75.X	1075	1070, 1095, 1523		1
Stroke (All)	9652	430.X, 431.X, 433.X, 434.X	I60.X, I61.X, I63.X		1081, 1086, 1491, 1583			
Ischemic stroke	4602	433.X, 434.X	I63.X		1081, 1583			
Haemorrhagic stroke	1981	430.X, 431.X	I60.X, I61.X		1491, 1086			
Abdominal aortic aneurysm	1094	441.3, 441.4	I71.3, I71.4	L19.4, L19.5				
Thoracic aortic aneurysm	347	441.1, 441.2	I71.1, I71.2					
Deep-vein thrombosis	9454	451.1	I80.2	L90.2	1094		7	
Pulmonary embolism	6148	415.1	I26.X		1093		5	
Peripheral vascular disease	3415	443.8, 443.9	I73.8, I73.9		1067			

Disease name	Number of cases*	Health conditions from electronic health records			Self-reported health conditions			
		ICD-9 Diagnosis	ICD-10 Diagnosis	Operative procedures (OPCS4)	Self-reported non-cancer illness code (20002)	Self-reported operation code (20004)	Code of vascular and bronchiolar conditions diagnosed by doctor (6152)	Code of vascular and heart problems diagnosed by doctor (6150)
Heart failure	6712	402.01, 402.11, 402.91, 404.01, 404.11, 404.91, 404.03, 404.13, 404.93, 428.X	I11.0, I13.0, I13.2, I50.X		1076, 1079, 1588			
Atrial fibrillation	16945	427.3	I48		1471			
Aortic stenosis	2244		I35.0, I35.2		1490			

Abbreviations: ICD: International Classification of Disease; OPCS4: Office of Population Censuses and Surveys Classification of Interventions and Procedures version 4.
*Include cases at baseline and during follow-up.

Table S2. Statistical power of the present study to detect a true odds ratio of 1.01, 1.02, 1.05 and 1.1 associated with 1mmol/mol-increment of HbA1c.

Disease	Cases (N)	Proportion of cases in the sample	Power (%) to detect a causal of 1mmol/mol increase in HbA1C:			
			OR = 1.01	OR = 1.02	OR = 1.05	OR = 1.1
Coronary artery disease	29278	7.96%	31.4%	83.6%	100.0%	100.0%
Stroke (all)	9652	2.62%	13.8%	41.1%	99.0%	100.0%
Haemorrhagic stroke	1981	0.54%	5.9%	12.2%	49.9%	96.9%
Ischemic stroke	4602	1.25%	8.8%	22.6%	84.4%	100.0%
Abdominal aortic aneurysm	1094	0.30%	4.8%	8.6%	30.7%	81.2%
Thoracic aortic aneurysm*	347	0.09%	3.7%	5.2%	12.7%	36.1%
Deep-vein thrombosis	9454	2.57%	13.6%	40.4%	98.8%	100.0%
Pulmonary embolism	6148	1.67%	10.4%	28.5%	92.9%	100.0%
Peripheral vascular disease	3415	0.93%	7.5%	17.9%	72.7%	99.9%
Heart failure	6712	1.83%	10.9%	30.6%	94.7%	100.0%
Atrial fibrillation	16945	4.61%	20.7%	62.4%	100.0%	100.0%
Aortic valve stenosis	2244	0.61%	6.2%	13.2%	54.9%	98.3%

*The outcome was underpowered (power < 80%) to detect any causal effect of 1mmol/mol increase in HbA1c with a size of OR≤1.1.

Table S3. Mendelian randomization estimates and 95% confidence intervals for cardiovascular outcomes per 2-fold increase in genetically predicted risk of type 2 diabetes mellitus. Analyses were performed in 367,703 UK Biobank participants of European ancestries, as well as in those without diabetes, and without diabetes or pre-diabetes**.**

**Disease**	**All participants**		**With diabetics excluded**		**With diabetics and pre-diabetics excluded**	
	**Odds Ratio (95% CI)**	**P-value**	**Odds Ratio (95% CI)**	**P-value**	**Odds Ratio (95% CI)**	**P-value**
Coronary artery disease	1.087 (1.062-1.113)	<0.001	1.021 (0.996-1.047)	0.098	1.012 (0.985-1.040)	0.378
Stroke (All)	1.049 (1.023-1.076)	<0.001	1.000 (0.973-1.027)	0.998	1.007 (0.977-1.038)	0.646
Ischaemic stroke	1.072 (1.033-1.112)	<0.001	1.006 (0.965-1.049)	0.77	1.007 (0.960-1.056)	0.768
Haemorrhagic stroke	1.005 (0.951-1.063)	0.861	0.997 (0.940-1.057)	0.908	1.015 (0.951-1.084)	0.648
Abdominal aortic aneurysm	0.982 (0.906-1.065)	0.668	0.979 (0.901-1.063)	0.608	0.976 (0.886-1.074)	0.612
Thoracic aortic aneurysm	0.973 (0.859-1.102)	0.668	1.010 (0.883-1.156)	0.882	1.011 (0.873-1.172)	0.88
Deep-vein thrombosis	1.050 (1.008-1.093)	0.018	1.028 (0.986-1.073)	0.198	1.032 (0.988-1.077)	0.159
Pulmonary embolism	1.013 (0.969-1.059)	0.564	1.003 (0.957-1.052)	0.886	1.008 (0.958-1.061)	0.75
Peripheral vascular disease	1.161 (1.112-1.212)	<0.001	1.047 (0.997-1.101)	0.067	1.041 (0.982-1.104)	0.174
Heart failure	1.087 (1.051-1.123)	<0.001	1.011 (0.973-1.051)	0.561	1.009 (0.967-1.052)	0.686
Atrial fibrillation	1.014 (0.989-1.040)	0.284	0.989 (0.963-1.017)	0.438	0.989 (0.961-1.018)	0.463
Aortic valve stenosis	1.133 (1.075-1.194)	<0.001	1.083 (1.023-1.146)	0.006	1.056 (0.990-1.127)	0.098

Table S4. Mendelian randomization estimates for cardiovascular outcomes per 2-fold increase in genetically predicted risk of type 2 diabetes mellitus in sensitivity analyses. Analyses were performed in 367,703 UK Biobank participants of European ancestries, using weighted median method and MR-Egger method.

Disease	Weighted median		MR-Egger			
	**Odds Ratio (95% CI)**	**P-value**	**Odds Ratio (95% CI)**	**P-value**	**Y-intercept**	**P-value**
Coronary artery disease	1.058 (1.026-1.092)	<0.001	1.016 (0.969-1.066)	0.505	0.007	0.002
Stroke (All)	1.044 (0.995-1.096)	0.081	1.028 (0.975-1.084)	0.304	0.002	0.394
Ischaemic stroke	1.055 (0.985-1.131)	0.124	1.053 (0.975-1.137)	0.188	0.002	0.606
Haemorrhagic stroke	0.936 (0.847-1.034)	0.192	0.930 (0.827-1.046)	0.228	0.008	0.143
Abdominal aortic aneurysm	0.887 (0.772-1.019)	0.089	0.887 (0.749-1.051)	0.168	0.011	0.182
Thoracic aortic aneurysm	0.938 (0.731-1.204)	0.614	0.926 (0.714-1.201)	0.563	0.005	0.671
Deep-vein thrombosis	1.021 (0.981-1.063)	0.299	0.985 (0.907-1.071)	0.731	0.007	0.09
Pulmonary embolism	0.990 (0.935-1.049)	0.738	0.963 (0.878-1.057)	0.432	0.005	0.226
Peripheral vascular disease	1.125 (1.039-1.219)	0.004	1.133 (1.036-1.239)	0.006	0.003	0.545
Heart failure	1.034 (0.986-1.084)	0.164	1.006 (0.939-1.077)	0.867	0.008	0.012
Atrial fibrillation	0.993 (0.958-1.029)	0.696	0.985 (0.935-1.038)	0.576	0.003	0.218
Aortic valve stenosis	1.080 (0.990-1.178)	0.083	1.046 (0.938-1.166)	0.422	0.008	0.1

Table S5. Cochran's Q statistics and p-values of heterogeneity test for Mendelian randomization estimates of type 2 diabetes mellitus on cardiovascular outcomes.

Disease	Entire Population		With diabetics excluded		With diabetics and pre-diabetics excluded	
	Cochran's Q	P-value	Cochran's Q	P-value	Cochran's Q	P-value
Coronary artery disease	638.862	<0.001	586.273	<0.001	524.663	<0.001
Stroke (All)	270.487	0.101	251.765	0.32	246.525	0.407
Ischaemic stroke	277.365	0.059	280.302	0.046	285.268	0.029
Haemorrhagic stroke	279.004	0.051	268.771	0.114	280.675	0.044
Abdominal aortic aneurysm	321.031	<0.001	282.789	0.037	256.83	0.245
Thoracic aortic aneurysm	247.307	0.394	261.531	0.185	268.47	0.117
Deep-vein thrombosis	687.709	<0.001	644.071	<0.001	548.314	<0.001
Pulmonary embolism	551.886	<0.001	523.416	<0.001	492.78	<0.001
Peripheral vascular disease	287.931	0.023	275.997	0.066	278.892	0.052
Heart failure	328.336	<0.001	325.601	<0.001	280.143	0.046
Atrial fibrillation	462.58	<0.001	444.402	<0.001	389.05	<0.001
Aortic valve stenosis	281.76	0.04	257.742	0.232	251.711	0.321

Table S6. Mendelian randomization estimates and 95% confidence intervals for cardiovascular outcomes per 1mmol/mol increase in genetically predicted HbA1c levels. Analyses were performed in 367,703 UK Biobank participants of European ancestries, as well as in those without diabetes, and without diabetes or pre-diabetes.

Disease	Entire Population		With diabetics and pre-diabetics excluded		With diabetics and pre-diabetics excluded	
	**Odds Ratio (95% CI)**	**P-value**	**Odds Ratio (95% CI)**	**P-value**	**Odds Ratio (95% CI)**	**P-value**
Coronary artery disease	1.036 (1.024-1.049)	<0.001	1.027 (1.014-1.039)	<0.001	1.007 (0.994-1.021)	0.27
Stroke (All)	1.027 (1.012-1.042)	<0.001	1.023 (1.007-1.040)	0.006	1.014 (0.995-1.032)	0.148
Ischaemic stroke	1.015 (0.994-1.038)	0.168	1.009 (0.985-1.033)	0.465	0.991 (0.965-1.019)	0.529
Haemorrhagic stroke	1.040 (1.007-1.073)	0.016	1.039 (1.005-1.075)	0.025	1.058 (1.020-1.097)	0.002
Abdominal aortic aneurysm	0.982 (0.940-1.025)	0.406	0.970 (0.925-1.017)	0.213	0.960 (0.906-1.017)	0.161
Thoracic aortic aneurysm	1.001 (0.931-1.076)	0.986	0.990 (0.916-1.070)	0.793	0.974 (0.896-1.060)	0.544
Deep-vein thrombosis	1.002 (0.982-1.023)	0.854	0.997 (0.975-1.019)	0.756	0.991 (0.968-1.014)	0.42
Pulmonary embolism	1.029 (1.005-1.054)	0.018	1.028 (1.002-1.054)	0.035	1.019 (0.991-1.048)	0.182
Peripheral vascular disease	1.036 (1.011-1.062)	0.005	1.025 (0.995-1.055)	0.099	0.999 (0.966-1.033)	0.951
Heart failure	1.017 (0.999-1.035)	0.063	0.997 (0.976-1.018)	0.754	0.981 (0.957-1.006)	0.129
Atrial fibrillation	0.999 (0.986-1.012)	0.908	0.991 (0.978-1.005)	0.225	0.988 (0.973-1.003)	0.118
Aortic valve stenosis	1.031 (1.000-1.064)	0.053	1.015 (0.982-1.050)	0.374	1.005 (0.968-1.043)	0.806

Table S7. Mendelian randomization estimates for cardiovascular outcomes per 1mmol/mol increase in genetically predicted HbA1c levels in supplementary analyses using 213 non-erythrocytic HbA1c variants.

Disease	Odds Ratio (95% CI)	P-value
Coronary artery disease	1.070 (1.050-1.091)	<0.001
Stroke (All)	1.032 (1.006-1.058)	0.015
Ischaemic stroke	1.031 (0.994-1.070)	0.098
Haemorrhagic stroke	1.037 (0.981-1.097)	0.198
Abdominal aortic aneurysm	1.025 (0.949-1.108)	0.53
Thoracic aortic aneurysm	0.950 (0.828-1.089)	0.458
Deep-vein thrombosis	0.990 (0.963-1.017)	0.47
Pulmonary embolism	0.998 (0.964-1.032)	0.896
Peripheral vascular disease	1.083 (1.036-1.133)	<0.001
Heart failure	1.026 (0.995-1.057)	0.097
Atrial fibrillation	0.983 (0.961-1.006)	0.141
Aortic valve stenosis	1.071 (1.014-1.131)	0.014

Table S8. Mendelian randomization estimates for cardiovascular outcomes per 1mmol/mol increase in genetically predicted HbA1c levels in sensitivity analyses. Analyses were performed in 367,703 UK Biobank participants of European ancestries, using weighted median method and MR-Egger method.

Disease	Weighted median		MR-Egger			
	**Odds Ratio (95% CI)**	**P-value**	**Odds Ratio (95% CI)**	**P-value**	**Y-intercept**	**P-value**
Coronary artery disease	1.045 (1.028-1.061)	<0.001	1.019 (0.998-1.041)	0.077	0.002	0.059
Stroke (All)	1.028 (1.004-1.054)	0.025	1.016 (0.989-1.043)	0.246	0.001	0.343
Ischaemic stroke	1.001 (0.967-1.036)	0.958	0.990 (0.953-1.030)	0.628	0.003	0.13
Haemorrhagic stroke	1.023 (0.971-1.078)	0.398	1.022 (0.966-1.081)	0.448	0.002	0.479
Abdominal aortic aneurysm	0.985 (0.916-1.058)	0.673	0.951 (0.881-1.027)	0.202	0.004	0.327
Thoracic aortic aneurysm	0.927 (0.816-1.052)	0.239	1.077 (0.945-1.226)	0.266	-0.01	0.185
Deep-vein thrombosis	0.988 (0.964-1.013)	0.338	0.974 (0.939-1.010)	0.157	0.004	0.067
Pulmonary embolism	1.029 (0.998-1.060)	0.064	1.007 (0.965-1.052)	0.744	0.003	0.233
Peripheral vascular disease	1.022 (0.982-1.064)	0.284	1.017 (0.973-1.064)	0.449	0.003	0.34
Heart failure	1.010 (0.981-1.040)	0.517	1.003 (0.972-1.036)	0.834	0.002	0.318
Atrial fibrillation	0.997 (0.979-1.017)	0.784	1.006 (0.983-1.030)	0.608	-0.001	0.487
Aortic valve stenosis	1.020 (0.971-1.072)	0.437	1.038 (0.982-1.098)	0.187	-0.001	0.769

Table S9. Cochran's Q statistics and p-values of heterogeneity test for Mendelian randomization estimates of HbA1c on cardiovascular outcomes.

Disease	Entire Population		With diabetics excluded		With diabetics and pre-diabetics excluded	
	Cochran's Q	P-value	Cochran's Q	P-value	Cochran's Q	P-value
Coronary artery disease	1057.748	<0.001	911.267	<0.001	830.687	<0.001
Stroke (All)	613.619	0.01	607.327	0.016	595.947	0.035
Ischaemic stroke	644.403	0.001	622.053	0.005	612.775	0.011
Haemorrhagic stroke	594.2	0.039	582.989	0.074	572.32	0.128
Abdominal aortic aneurysm	626.071	0.004	630.092	0.003	625.811	0.004
Thoracic aortic aneurysm	546.148	0.36	570.275	0.141	568.392	0.154
Deep-vein thrombosis	1178.211	<0.001	1127.194	<0.001	1008.408	<0.001
Pulmonary embolism	1046.702	<0.001	1003.721	<0.001	955.85	<0.001
Peripheral vascular disease	632.662	0.002	632.604	0.002	611.626	0.012
Heart failure	630.673	0.003	626.037	0.004	659.092	<0.001
Atrial fibrillation	784.411	<0.001	753.166	<0.001	714.868	<0.001
Aortic valve stenosis	647.875	0.001	583.057	0.074	565.814	0.172

Table S10. Genetic instruments of 243 T2DM variants and details of associations with T2DM.

SNP	Effect allele	Alternative allele	Frequency of effect allele	β-coefficient with T2DM		
				β-coefficient	Standard error	P-value
rs184660829	T	C	1	-2.1	0.37	2.50E-08
rs1127215	T	C	0.42	-0.047	0.0064	2.30E-13
rs1493694	T	C	0.11	0.084	0.01	2.10E-16
rs145904381	T	C	0.99	0.17	0.031	2.20E-08
rs539515	A	C	0.8	-0.051	0.008	1.20E-10
rs12048743	C	G	0.56	-0.038	0.0064	4.40E-09
rs9430095	C	G	0.49	0.036	0.0064	2.30E-08
rs340874	T	C	0.44	-0.068	0.0064	5.60E-26
rs2820446	C	G	0.71	0.057	0.007	3.70E-16
rs348330	A	G	0.64	-0.051	0.0067	3.90E-14
rs291367	A	G	0.37	-0.044	0.007	6.10E-10
rs3768321	T	G	0.2	0.085	0.008	1.30E-26
rs58432198	T	C	0.12	-0.065	0.01	1.80E-10
rs12140153	T	G	0.095	-0.064	0.011	1.20E-08
rs7903146	T	C	0.29	0.31	0.0069	0.00E+00
rs72631105	A	G	0.19	0.044	0.0083	8.00E-08
rs11257655	T	C	0.22	0.09	0.0076	3.70E-32
rs2280141	T	G	0.52	0.047	0.0063	2.00E-13
rs2642588	T	G	0.3	-0.052	0.0069	6.30E-14
rs703972	C	G	0.47	-0.071	0.0064	2.50E-28
rs11202627	T	C	0.15	0.044	0.0089	7.60E-07
rs10882101	T	C	0.59	0.11	0.0064	1.60E-62
rs67232546	T	C	0.21	0.056	0.008	1.40E-12
rs141521721	A	C	0.024	0.12	0.021	2.80E-08
rs5213	T	C	0.64	-0.071	0.0066	1.90E-26
rs4929965	A	G	0.38	0.07	0.0067	4.80E-25
rs4923543	A	G	0.33	0.026	0.0067	1.00E-04
rs2237895	A	C	0.57	-0.093	0.0066	3.60E-44
rs145678014	T	G	0.043	-0.11	0.016	1.10E-11
rs2767036	A	C	0.71	-0.039	0.0069	2.50E-08
rs1061810	A	C	0.29	0.05	0.007	8.50E-13
rs7115753	A	G	0.45	0.038	0.0064	4.80E-09
rs7124681	A	C	0.41	0.037	0.0064	6.40E-09
rs1783541	T	C	0.2	0.061	0.008	1.40E-14
rs11820019	T	C	0.97	0.14	0.021	1.00E-11
rs77464186	A	C	0.84	0.11	0.0088	2.30E-33
rs10830963	C	G	0.72	-0.099	0.0071	1.50E-43
rs1426371	A	G	0.26	-0.05	0.0073	1.10E-11
rs34965774	A	G	0.14	0.054	0.0092	3.50E-09
rs56348580	C	G	0.31	-0.062	0.0069	3.80E-19

SNP	Effect allele	Alternative allele	Frequency of effect allele	β-coefficient with T2DM		
				β-coefficient	Standard error	P-value
rs4148856	C	G	0.78	0.049	0.0077	2.20E-10
rs7978610	C	G	0.33	-0.034	0.0067	5.40E-07
rs2066827	T	G	0.77	-0.044	0.0081	3.50E-08
rs12811407	A	G	0.33	0.049	0.007	2.40E-12
rs718314	A	G	0.75	-0.047	0.0073	1.10E-10
rs10842994	T	C	0.19	-0.074	0.0081	2.50E-20
rs76895963	T	G	0.98	0.48	0.027	5.30E-70
rs2258238	A	T	0.9	-0.11	0.011	2.00E-25
rs1796330	C	G	0.43	-0.049	0.0064	3.20E-14
rs2197973	T	C	0.54	0.035	0.0063	4.40E-08
rs77864822	A	G	0.93	0.073	0.013	2.20E-08
rs7987740	T	C	0.61	0.036	0.0065	4.10E-08
rs34584161	A	G	0.76	0.048	0.0075	2.90E-10
rs11842871	T	G	0.27	-0.042	0.0073	1.50E-08
rs576674	A	G	0.83	-0.053	0.0086	6.80E-10
rs963740	A	T	0.71	0.039	0.007	2.60E-08
rs9537803	T	C	0.72	-0.034	0.007	1.30E-06
rs9563615	A	T	0.71	0.042	0.007	3.90E-09
rs1359790	A	G	0.28	-0.083	0.0071	5.70E-31
rs62007683	T	G	0.35	-0.037	0.0067	3.80E-08
rs17122772	C	G	0.77	-0.043	0.0077	2.00E-08
rs17522122	T	G	0.47	0.038	0.0064	4.00E-09
rs8017808	T	G	0.26	-0.041	0.0073	2.60E-08
rs17836088	C	G	0.22	0.058	0.0077	9.70E-14
rs8010382	A	G	0.58	-0.038	0.0066	8.10E-09
rs34715063	T	C	0.88	-0.076	0.01	3.30E-14
rs11070332	A	G	0.36	0.049	0.0066	1.30E-13
rs2456530	T	C	0.13	0.056	0.0096	4.70E-09
rs528350911	C	G	0.99	-0.24	0.043	2.10E-08
rs117483894	A	G	0.96	-0.093	0.017	3.90E-08
rs8037894	C	G	0.43	-0.047	0.0064	3.70E-13
rs7178762	T	C	0.54	-0.039	0.0063	7.00E-10
rs4776970	A	T	0.64	0.039	0.0066	6.20E-09
rs13737	T	G	0.24	-0.046	0.0075	7.30E-10
rs1005752	A	C	0.72	0.079	0.007	5.70E-29
rs4932265	T	C	0.27	0.065	0.0071	7.20E-20
rs12910825	A	G	0.64	-0.053	0.0066	2.40E-15
rs8046545	A	G	0.64	-0.037	0.0066	2.30E-08
rs6600191	T	C	0.82	0.061	0.0085	7.00E-13
rs11642430	C	G	0.6	-0.042	0.0065	1.20E-10
rs3751837	T	C	0.22	0.044	0.0077	1.70E-08
rs1421085	T	C	0.58	-0.12	0.0064	2.40E-78
rs862320	T	C	0.42	-0.042	0.0064	5.10E-11

SNP	Effect allele	Alternative allele	Frequency of effect allele	β-coefficient with T2DM		
				β-coefficient	Standard error	P-value
rs72802342	A	C	0.077	-0.13	0.012	1.30E-27
rs2925979	T	C	0.3	0.053	0.0069	2.10E-14
rs12920022	A	T	0.16	0.053	0.009	2.90E-09
rs4925109	A	G	0.32	0.048	0.0068	3.90E-12
rs71372253	T	C	0.94	-0.073	0.013	4.30E-08
rs10908278	A	T	0.52	-0.074	0.0064	3.10E-30
rs1377807	C	G	0.31	0.057	0.0068	5.70E-17
rs34855406	C	G	0.28	0.05	0.0071	3.20E-12
rs35895680	A	C	0.32	-0.055	0.0069	3.80E-15
rs569511541	A	G	1	-2	0.36	1.50E-08
rs60276348	T	C	0.14	0.052	0.0095	2.90E-08
rs61676547	C	G	0.19	0.055	0.0081	1.00E-11
rs1641523	T	C	0.57	-0.028	0.0064	1.30E-05
rs7222481	C	G	0.32	0.039	0.0068	1.70E-08
rs62080313	T	C	0.88	-0.056	0.0098	9.10E-09
rs72926932	A	C	0.92	-0.083	0.011	3.60E-13
rs17684074	C	G	0.26	-0.041	0.0073	3.50E-08
rs9957145	A	G	0.17	-0.05	0.0086	6.70E-09
rs523288	A	T	0.76	-0.056	0.0074	7.50E-14
rs12454712	T	C	0.61	0.049	0.0067	5.10E-13
rs7240767	T	C	0.62	-0.037	0.0065	2.00E-08
rs3111316	A	G	0.59	0.046	0.0065	1.60E-12
rs8107974	A	T	0.92	-0.093	0.012	6.30E-15
rs10406327	C	G	0.52	0.035	0.0064	4.60E-08
rs429358	T	C	0.85	0.08	0.0092	1.80E-18
rs10406431	A	G	0.56	0.059	0.0065	2.50E-19
rs3810291	A	G	0.67	0.046	0.0068	1.20E-11
rs7249758	A	G	0.2	0.045	0.008	1.20E-08
rs75253922	T	C	0.81	-0.046	0.0082	2.20E-08
rs4804833	A	G	0.39	0.047	0.0066	1.10E-12
rs562386202	A	G	1	-1.2	0.21	4.20E-08
rs11688682	C	G	0.27	-0.058	0.0075	1.40E-14
rs35999103	T	C	0.15	0.052	0.0091	8.30E-09
rs13426680	A	G	0.94	0.082	0.013	6.40E-10
rs3772071	T	C	0.71	0.048	0.007	1.60E-11
rs10195252	T	C	0.59	0.06	0.0064	1.60E-20
rs11680058	A	G	0.86	0.058	0.01	1.30E-08
rs113414093	A	G	0.061	0.08	0.016	6.40E-07
rs2972144	A	G	0.36	-0.094	0.0066	7.90E-46
rs17802463	T	G	0.27	-0.039	0.0071	3.50E-08
rs1260326	T	C	0.39	-0.067	0.0065	1.30E-24
rs62107261	T	C	0.95	0.11	0.016	1.80E-11
rs80147536	A	T	0.9	0.13	0.011	2.70E-30

SNP	Effect allele	Alternative allele	Frequency of effect allele	β-coefficient with T2DM		
				β-coefficient	Standard error	P-value
rs6545714	A	G	0.61	-0.037	0.0065	1.70E-08
rs243024	A	G	0.46	0.058	0.0063	4.40E-20
rs2249105	A	G	0.63	0.053	0.0066	1.20E-15
rs79046683	T	G	0.0045	0.74	0.14	1.80E-07
rs13041756	T	C	0.89	-0.058	0.01	1.30E-08
rs2268078	A	G	0.66	0.043	0.0067	2.90E-10
rs1800961	T	C	0.035	0.16	0.017	3.20E-20
rs6063048	A	G	0.28	-0.047	0.0071	5.80E-11
rs11699802	T	C	0.46	-0.043	0.0064	2.50E-11
rs34454109	A	T	0.77	0.044	0.0076	8.80E-09
rs6070625	C	G	0.48	-0.044	0.0063	3.20E-12
rs59944054	A	G	0.24	0.033	0.0079	2.90E-05
rs6518681	A	G	0.086	-0.083	0.012	9.60E-13
rs117001013	T	C	0.088	-0.065	0.011	1.50E-08
rs5758223	A	G	0.72	0.038	0.007	4.60E-08
rs738408	T	C	0.23	0.049	0.0076	1.80E-10
rs1801645	T	C	0.72	-0.048	0.0074	1.50E-10
rs11708067	A	G	0.77	0.089	0.0076	1.30E-31
rs11709077	A	G	0.12	-0.11	0.0098	1.60E-27
rs649961	T	C	0.47	0.038	0.0063	1.30E-09
rs9828772	C	G	0.9	0.059	0.011	4.20E-08
rs62271373	A	T	0.055	0.088	0.014	1.00E-09
rs13065698	A	G	0.6	0.039	0.0065	3.60E-09
rs7629630	A	T	0.86	0.051	0.0091	2.20E-08
rs9873618	A	G	0.29	-0.066	0.007	8.50E-21
rs2872246	A	C	0.45	0.036	0.0063	1.80E-08
rs6780171	A	T	0.31	0.11	0.0068	2.50E-58
rs3887925	T	C	0.55	0.055	0.0064	1.40E-17
rs4686471	T	C	0.39	-0.06	0.0065	3.10E-20
rs35352848	T	C	0.79	0.071	0.0079	9.50E-20
rs11926707	T	C	0.37	-0.038	0.0066	1.50E-08
rs4688760	T	C	0.68	0.043	0.0069	4.50E-10
rs2581787	T	G	0.56	0.036	0.0064	3.00E-08
rs76263492	T	G	0.045	0.091	0.016	6.30E-09
rs3774723	A	G	0.16	-0.065	0.0089	2.00E-13
rs9860730	A	G	0.7	0.055	0.007	7.40E-15
rs13085136	T	C	0.072	-0.074	0.013	1.40E-08
rs2272163	A	C	0.38	-0.037	0.0065	1.20E-08
rs1580278	A	C	0.53	-0.041	0.0064	2.90E-10
rs1296328	A	C	0.45	0.035	0.0064	4.30E-08
rs7669833	A	T	0.3	-0.054	0.007	1.80E-14
rs28819812	A	C	0.32	-0.04	0.0072	2.70E-08
rs12640250	A	C	0.29	-0.039	0.0071	4.50E-08

SNP	Effect allele	Alternative allele	Frequency of effect allele	β-coefficient with T2DM		
				β-coefficient	Standard error	P-value
rs56337234	T	C	0.5	-0.057	0.0066	1.40E-17
rs58730668	T	C	0.86	0.068	0.0092	1.00E-13
rs362307	T	C	0.077	0.074	0.012	1.10E-09
rs10938398	A	G	0.43	0.044	0.0064	4.90E-12
rs2102278	A	G	0.68	-0.038	0.0069	4.50E-08
rs10937721	C	G	0.59	0.087	0.0065	1.60E-40
rs1531583	T	G	0.046	0.11	0.015	1.20E-12
rs12642790	A	G	0.34	0.042	0.0067	5.70E-10
rs1903002	C	G	0.5	-0.036	0.0064	3.00E-08
rs6821438	A	G	0.53	0.042	0.0063	5.40E-11
rs138337556	A	G	1	-0.53	0.074	8.90E-13
rs115505614	T	C	0.05	0.17	0.015	1.70E-29
rs329122	A	G	0.43	0.037	0.0064	9.20E-09
rs78408340	T	C	0.0062	-0.38	0.047	8.70E-16
rs3934712	T	C	0.79	-0.039	0.0079	6.30E-07
rs6884702	A	G	0.61	-0.038	0.0065	5.80E-09
rs3811978	A	G	0.83	-0.053	0.0085	4.20E-10
rs702634	A	G	0.69	0.051	0.0069	2.10E-13
rs465002	T	C	0.74	0.073	0.0073	3.80E-23
rs4976033	A	G	0.59	-0.035	0.0065	1.00E-07
rs2307111	T	C	0.61	0.053	0.0065	3.30E-16
rs4457053	A	G	0.7	-0.059	0.0069	1.40E-17
rs1316776	A	C	0.35	-0.046	0.0066	3.50E-12
rs7719891	A	G	0.74	-0.04	0.0072	2.90E-08
rs4946812	A	G	0.33	-0.039	0.0068	1.00E-08
rs11759026	A	G	0.77	-0.067	0.0075	1.30E-18
rs2800733	A	G	0.72	0.051	0.007	3.70E-13
rs9494624	A	G	0.29	0.041	0.007	7.60E-09
rs2982521	A	T	0.38	0.034	0.0065	3.00E-07
rs474513	A	G	0.52	0.039	0.0063	1.00E-09
rs4709746	T	C	0.13	-0.056	0.0096	5.00E-09
rs7756992	A	G	0.73	-0.14	0.007	3.00E-87
rs601945	A	G	0.82	-0.08	0.0085	2.70E-21
rs77136196	T	C	0.041	0.086	0.017	2.00E-07
rs34298980	T	C	0.5	0.04	0.0066	1.20E-09
rs6458354	T	C	0.71	-0.051	0.007	3.70E-13
rs3798519	A	C	0.82	-0.058	0.0082	1.10E-12
rs555402748	T	C	0.0004	1.3	0.24	4.60E-08
rs9379084	A	G	0.11	-0.097	0.011	2.30E-20
rs11496066	T	C	0.82	0.047	0.0083	1.20E-08
rs39328	T	C	0.43	0.036	0.0064	3.00E-08
rs6976111	A	C	0.31	0.042	0.0073	1.50E-08
rs1562396	A	G	0.68	-0.058	0.0069	7.60E-17

SNP	Effect allele	Alternative allele	Frequency of effect allele	β-coefficient with T2DM		
				β-coefficient	Standard error	P-value
rs62492368	A	G	0.31	0.044	0.0069	1.50E-10
rs10228066	T	C	0.54	0.066	0.0063	1.90E-25
rs6459733	C	G	0.33	-0.058	0.0068	3.90E-17
rs4279506	C	G	0.39	-0.039	0.0066	5.70E-09
rs1708302	T	C	0.49	-0.092	0.0063	4.20E-48
rs917195	T	C	0.23	-0.051	0.0077	5.60E-11
rs878521	A	G	0.25	0.057	0.0074	1.60E-14
rs57327348	A	T	0.78	0.053	0.0079	2.10E-11
rs12680028	C	G	0.53	0.035	0.0063	3.10E-08
rs3802177	A	G	0.31	-0.11	0.0069	6.30E-55
rs17772814	A	G	0.085	-0.078	0.013	5.00E-10
rs1561927	T	C	0.73	-0.043	0.0071	1.90E-09
rs4977213	T	C	0.63	-0.051	0.0067	4.40E-14
rs10096633	T	C	0.12	-0.07	0.0098	8.70E-13
rs10954772	T	C	0.31	0.041	0.0068	2.30E-09
rs13262861	A	C	0.17	-0.094	0.0087	1.80E-27
rs10097617	T	C	0.48	0.051	0.0063	1.10E-15
rs149364428	A	G	0.01	0.24	0.034	1.90E-12
rs17689007	A	G	0.47	-0.048	0.0064	1.70E-13
rs505922	T	C	0.67	-0.046	0.0067	5.40E-12
rs28505901	A	G	0.25	-0.076	0.0081	2.60E-21
rs7022807	A	G	0.6	-0.04	0.0064	3.60E-10
rs7867635	T	C	0.59	-0.036	0.0065	4.10E-08
rs10811660	A	G	0.17	-0.16	0.0086	6.60E-79
rs1412234	T	C	0.68	-0.043	0.0068	2.50E-10
rs12001437	T	C	0.63	-0.041	0.0065	3.70E-10
rs10974438	A	C	0.64	-0.051	0.0066	1.60E-14
rs11137820	C	G	0.58	0.035	0.0064	3.60E-08
rs17791513	A	G	0.93	0.1	0.013	2.90E-14
rs2796441	A	G	0.41	-0.066	0.0065	8.50E-24
rs55653563	A	C	0.73	0.043	0.0072	3.20E-09

Table S11. Genetic instruments of 536 HbA1c variants and details of associations with HbA1c. Genetic variants were screened for their associations with erythrocytic traits, including haemoglobin concentration, red blood cell count, haematocrit, mean corpuscular volume, mean corpuscular haemoglobin concentration, mean corpuscular haemoglobin, red cell distribution width, reticulocyte count, reticulocyte fraction of red cells, immature fraction of reticulocytes, high light scatter percentage of red cells and high light scatter reticulocyte count. Erythrocytic variants are variants associated with any of these traits with a P-value<0.001.

SNP	Effect allele	Alternative allele	Frequency of effect allele	β-coefficient with HbA1c (mmol/mol)			R2	F-statistics
				β-coefficient	Standard error	P-value		
Non-erythrocytic variants:								
rs13431652	T	C	0.310	0.404	0.011	0.00E+00	5.81E-04	237.67
rs1004558	C	T	0.178	-0.448	0.011	1.40E-320	4.81E-04	196.64
2:169809199	TTG	T	0.442	-0.284	0.011	2.90E-180	3.36E-04	137.66
rs10830963	C	G	0.274	-0.240	0.011	4.10E-128	1.89E-04	77.44
rs7903146	C	T	0.286	-0.197	0.011	7.70E-91	1.34E-04	54.62
rs11708067	A	G	0.247	0.186	0.011	1.40E-75	1.11E-04	45.35
rs12611808	T	C	0.321	-0.164	0.011	1.40E-64	9.42E-05	38.52
rs1604038	C	T	0.285	0.164	0.011	1.70E-60	8.80E-05	35.99
rs71430652	T	C	0.133	-0.208	0.011	7.50E-58	8.50E-05	34.78
rs76323047	A	G	0.118	-0.219	0.011	6.00E-57	8.34E-05	34.12
rs10811660	G	A	0.175	0.175	0.011	1.80E-53	7.76E-05	31.75
rs61944004	G	A	0.218	-0.142	0.011	2.30E-42	6.11E-05	24.99
rs368865	A	G	0.274	-0.131	0.011	2.40E-40	5.78E-05	23.63
rs138917529	A	T	0.015	0.459	0.033	7.70E-36	5.36E-05	21.91
rs28378473	T	C	0.267	-0.120	0.011	4.70E-34	4.90E-05	20.02
rs12712928	G	C	0.170	-0.142	0.011	6.30E-31	4.46E-05	18.25
rs36020935	G	T	0.197	-0.131	0.011	4.70E-21	4.39E-05	17.94
rs73032206	A	G	0.032	-0.284	0.022	3.70E-29	4.20E-05	17.16
rs11257655	C	T	0.208	-0.120	0.011	2.40E-29	4.14E-05	16.93
rs12459419	C	T	0.327	0.109	0.011	4.60E-29	4.10E-05	16.75
rs11564725	C	T	0.235	-0.109	0.011	1.00E-27	3.95E-05	16.15
rs115128825	C	A	0.020	-0.328	0.033	5.60E-26	3.66E-05	14.95
rs17850433	T	C	0.012	-0.426	0.044	5.30E-26	3.62E-05	14.82
rs56233660	G	A	0.085	-0.164	0.011	5.80E-24	3.40E-05	13.91

SNP	Effect allele	Alternative allele	Frequency of effect allele	β-coefficient with HbA1c (mmol/mol)			R2	F-statistics
				β-coefficient	Standard error	P-value		
rs11602873	A	T	0.159	0.120	0.011	2.10E-24	3.40E-05	13.91
rs2389615	T	C	0.144	-0.131	0.011	1.20E-21	3.34E-05	13.68
rs142394825	G	C	0.014	0.383	0.044	1.40E-22	3.32E-05	13.58
rs149966	G	T	0.263	0.098	0.011	7.00E-14	3.31E-05	13.53
rs11531378	T	C	0.300	-0.098	0.011	3.90E-23	3.23E-05	13.20
rs6980437	T	G	0.476	-0.087	0.011	8.50E-23	3.19E-05	13.04
rs860262	C	A	0.497	0.087	0.011	1.20E-22	3.14E-05	12.83
rs11187140	G	A	0.361	0.087	0.011	5.70E-22	3.06E-05	12.53
rs146302237	A	AT	0.134	0.120	0.011	3.70E-21	3.02E-05	12.34
rs10407429	G	A	0.427	0.087	0.011	9.30E-22	3.01E-05	12.32
rs11540050	G	T	0.050	0.186	0.022	9.40E-20	2.73E-05	11.18
rs78152251	T	C	0.250	0.087	0.011	2.50E-19	2.67E-05	10.92
rs45499402	G	C	0.114	-0.120	0.011	1.20E-18	2.54E-05	10.38
rs4418728	G	T	0.449	0.077	0.011	1.30E-18	2.54E-05	10.37
rs61990729	C	T	0.229	0.087	0.011	1.30E-18	2.53E-05	10.37
rs143667358	TATACATACATAC	T	0.195	-0.098	0.011	3.30E-18	2.53E-05	10.35
rs576674	G	A	0.166	0.109	0.011	1.80E-18	2.52E-05	10.31
rs2237895	A	C	0.415	-0.077	0.011	1.60E-18	2.51E-05	10.28
rs4713574	C	G	0.449	-0.077	0.011	3.20E-17	2.50E-05	10.22
rs113204516	A	G	0.050	0.175	0.022	8.40E-18	2.49E-05	10.18
rs2808454	A	T	0.459	-0.077	0.011	6.00E-18	2.45E-05	10.03
rs143945190	A	G	0.008	0.426	0.055	5.20E-15	2.42E-05	9.91
rs17168486	C	T	0.174	-0.098	0.011	2.70E-17	2.36E-05	9.67
rs6777684	A	G	0.392	-0.077	0.011	5.00E-17	2.33E-05	9.51
rs71274822	C	CT	0.202	0.087	0.011	4.30E-17	2.32E-05	9.50
rs28663084	G	A	0.330	0.077	0.011	4.80E-17	2.32E-05	9.49
rs74598960	T	C	0.061	0.153	0.022	1.00E-15	2.24E-05	9.15

SNP	Effect allele	Alternative allele	Frequency of effect allele	β-coefficient with HbA1c (mmol/mol)			R2	F-statistics
				β-coefficient	Standard error	P-value		
rs17508261	T	C	0.122	-0.109	0.011	2.10E-16	2.21E-05	9.05
rs75367758	G	A	0.049	0.164	0.022	5.40E-16	2.16E-05	8.81
rs12147688	G	T	0.257	-0.087	0.011	8.00E-16	2.15E-05	8.80
rs61138219	C	T	0.153	0.098	0.011	1.00E-15	2.12E-05	8.68
rs238763	T	A	0.407	0.077	0.011	1.70E-15	2.10E-05	8.59
rs3789586	C	T	0.153	0.098	0.011	1.40E-15	2.10E-05	8.57
rs147001976	G	A	0.035	0.197	0.022	8.40E-15	2.09E-05	8.54
rs117056999	C	T	0.035	-0.197	0.022	2.20E-14	2.08E-05	8.50
rs3217860	A	G	0.248	-0.077	0.011	3.00E-15	2.06E-05	8.43
rs10873398	G	A	0.353	0.077	0.011	3.40E-15	2.04E-05	8.36
rs35474292	T	A	0.327	-0.077	0.011	3.40E-14	2.01E-05	8.21
rs79509806	C	T	0.025	-0.219	0.033	8.50E-15	1.99E-05	8.14
rs13165038	T	C	0.328	-0.077	0.011	9.00E-15	1.98E-05	8.11
rs9366739	G	A	0.087	-0.120	0.011	8.20E-15	1.97E-05	8.06
rs1560980	G	C	0.042	-0.175	0.022	1.20E-14	1.95E-05	7.99
rs9903102	A	C	0.141	0.098	0.011	1.40E-14	1.93E-05	7.90
rs599134	C	G	0.225	-0.077	0.011	1.80E-14	1.93E-05	7.88
rs6973494	C	A	0.485	-0.066	0.011	4.10E-14	1.92E-05	7.86
rs4473913	C	T	0.499	0.066	0.011	2.60E-14	1.90E-05	7.77
rs2390730	C	T	0.139	-0.098	0.011	3.40E-14	1.89E-05	7.74
rs2812541	A	C	0.459	-0.066	0.011	3.30E-14	1.89E-05	7.74
rs116710147	C	T	0.013	-0.295	0.044	1.20E-13	1.85E-05	7.55
rs28565268	T	C	0.070	-0.131	0.022	2.10E-10	1.83E-05	7.48
rs9302377	C	T	0.479	-0.066	0.011	1.90E-12	1.82E-05	7.44
rs7151822	T	C	0.248	0.077	0.011	1.40E-13	1.80E-05	7.38
rs17036143	G	T	0.131	-0.098	0.011	1.40E-13	1.80E-05	7.36
rs636089	T	C	0.386	-0.066	0.011	1.70E-13	1.79E-05	7.31
rs11768656	C	T	0.036	0.175	0.022	1.70E-13	1.77E-05	7.26

SNP	Effect allele	Alternative allele	Frequency of effect allele	β-coefficient with HbA1c (mmol/mol)			R2	F-statistics
				β-coefficient	Standard error	P-value		
rs4820059	G	A	0.406	0.066	0.011	1.90E-13	1.77E-05	7.26
rs12969025	C	T	0.343	0.066	0.011	2.00E-13	1.77E-05	7.26
rs28659953	T	C	0.391	-0.066	0.011	3.40E-13	1.77E-05	7.24
rs72807721	C	T	0.021	-0.230	0.033	2.10E-13	1.76E-05	7.21
rs258222	C	T	0.458	0.066	0.011	2.70E-13	1.76E-05	7.18
rs117473279	G	A	0.015	0.262	0.033	2.40E-12	1.75E-05	7.15
rs184499898	A	G	0.011	-0.317	0.044	1.10E-12	1.75E-05	7.15
rs1509896	G	C	0.092	0.109	0.011	5.50E-12	1.74E-05	7.13
6:29748690	A	G	0.320	-0.066	0.011	4.80E-13	1.73E-05	7.09
rs882834	C	A	0.140	-0.087	0.011	3.70E-13	1.73E-05	7.07
rs112769772	T	G	0.030	0.186	0.022	4.80E-12	1.70E-05	6.94
rs11597148	G	T	0.186	-0.077	0.011	2.30E-12	1.69E-05	6.91
rs757110	C	A	0.358	0.066	0.011	8.40E-13	1.68E-05	6.86
rs3757970	A	G	0.379	-0.066	0.011	9.80E-13	1.66E-05	6.80
rs73919194	T	A	0.066	0.131	0.022	2.80E-12	1.65E-05	6.77
rs7317848	T	A	0.068	-0.120	0.022	1.80E-08	1.65E-05	6.76
rs150065473	C	T	0.008	-0.350	0.055	1.50E-11	1.64E-05	6.69
rs13318260	A	G	0.435	-0.066	0.011	1.90E-12	1.63E-05	6.69
rs56087308	A	G	0.034	-0.175	0.022	1.80E-12	1.63E-05	6.67
rs72814215	G	A	0.013	0.273	0.044	7.70E-11	1.62E-05	6.62
rs7546017	C	T	0.252	-0.077	0.011	2.00E-12	1.62E-05	6.61
rs543159	C	A	0.472	0.066	0.011	4.10E-12	1.58E-05	6.48
rs117777577	C	T	0.024	-0.197	0.033	7.10E-11	1.58E-05	6.45
rs35849071	CA	C	0.092	-0.109	0.022	5.50E-09	1.58E-05	6.44
rs372855300	A	C	0.164	0.087	0.011	3.10E-11	1.58E-05	6.44
rs9759557	G	C	0.274	-0.066	0.011	4.40E-12	1.57E-05	6.44
rs2284344	G	C	0.484	0.066	0.011	5.30E-12	1.57E-05	6.43
rs10743139	G	A	0.051	0.142	0.022	4.60E-12	1.56E-05	6.40

SNP	Effect allele	Alternative allele	Frequency of effect allele	β-coefficient with HbA1c (mmol/mol)			R2	F-statistics
				β-coefficient	Standard error	P-value		
rs11392438	T	TA	0.260	0.066	0.011	3.60E-11	1.55E-05	6.34
rs925095	C	T	0.392	0.066	0.011	9.40E-12	1.55E-05	6.33
rs1579238	A	G	0.241	0.066	0.011	7.10E-12	1.54E-05	6.29
rs117720468	G	C	0.024	-0.197	0.033	1.00E-11	1.52E-05	6.24
rs1248882	C	T	0.345	-0.066	0.011	1.10E-11	1.52E-05	6.21
rs2168101	C	A	0.311	0.066	0.011	4.00E-11	1.51E-05	6.20
rs61735313	G	T	0.022	0.208	0.033	1.00E-11	1.51E-05	6.19
rs73082037	C	A	0.295	0.066	0.011	1.30E-11	1.51E-05	6.17
rs55859594	C	T	0.069	-0.120	0.022	6.70E-11	1.50E-05	6.13
rs17462188	C	T	0.309	-0.066	0.011	1.90E-11	1.50E-05	6.12
rs76957877	C	T	0.256	0.066	0.011	2.20E-11	1.48E-05	6.05
rs2071053	A	G	0.266	-0.066	0.011	2.10E-11	1.47E-05	6.01
rs920356	T	G	0.272	0.066	0.011	2.40E-11	1.47E-05	6.00
rs117817570	G	A	0.012	0.273	0.044	1.10E-09	1.44E-05	5.88
rs10863950	C	T	0.186	-0.077	0.011	3.60E-11	1.43E-05	5.87
rs201539436	TA	T	0.185	-0.077	0.011	8.70E-11	1.43E-05	5.85
rs374017936	T	TTA	0.263	-0.066	0.011	4.70E-11	1.43E-05	5.84
rs28483077	A	G	0.167	-0.077	0.011	4.80E-11	1.43E-05	5.83
rs7603054	G	A	0.283	0.066	0.011	4.40E-11	1.42E-05	5.81
rs73038369	G	T	0.406	0.055	0.011	5.10E-11	1.42E-05	5.80
rs2244566	A	C	0.156	0.077	0.011	7.60E-11	1.42E-05	5.79
rs4779054	A	G	0.368	-0.055	0.011	1.10E-10	1.40E-05	5.72
rs2286480	C	T	0.327	-0.066	0.011	7.60E-11	1.40E-05	5.72
rs117108573	C	T	0.081	0.109	0.022	9.40E-10	1.40E-05	5.71
rs138157637	A	G	0.048	0.131	0.022	2.20E-10	1.39E-05	5.67
rs492699	T	C	0.225	-0.066	0.011	4.70E-10	1.38E-05	5.64
rs7399581	T	G	0.115	-0.087	0.011	1.20E-10	1.36E-05	5.56
rs55828957	A	T	0.059	0.120	0.022	3.60E-10	1.36E-05	5.55

SNP	Effect allele	Alternative allele	Frequency of effect allele	β-coefficient with HbA1c (mmol/mol)			R2	F-statistics
				β-coefficient	Standard error	P-value		
rs12985264	G	A	0.323	-0.066	0.011	7.70E-09	1.33E-05	5.45
rs2583934	G	T	0.138	-0.077	0.011	2.20E-10	1.33E-05	5.42
rs2786487	G	C	0.440	-0.055	0.011	2.00E-10	1.33E-05	5.42
rs144192755	C	A	0.012	0.262	0.044	1.00E-09	1.31E-05	5.36
rs2248933	G	T	0.236	0.066	0.011	2.90E-10	1.31E-05	5.36
rs13327021	C	T	0.350	-0.055	0.011	2.60E-10	1.31E-05	5.35
rs1977383	T	C	0.450	-0.055	0.011	4.60E-10	1.29E-05	5.29
rs10758593	G	A	0.396	-0.055	0.011	3.20E-10	1.29E-05	5.28
rs35781149	C	A	0.035	0.153	0.022	5.30E-10	1.29E-05	5.27
rs13192435	T	C	0.025	-0.175	0.033	5.50E-10	1.26E-05	5.16
rs7222851	T	C	0.486	0.055	0.011	6.00E-10	1.26E-05	5.16
rs55689310	G	A	0.018	-0.208	0.033	6.70E-09	1.26E-05	5.15
rs6772129	A	G	0.292	0.066	0.011	6.60E-10	1.25E-05	5.13
rs141995849	G	A	0.063	-0.109	0.022	1.90E-09	1.25E-05	5.12
rs11070339	A	G	0.421	-0.055	0.011	9.00E-10	1.24E-05	5.08
rs7681002	A	G	0.484	0.055	0.011	9.00E-10	1.24E-05	5.06
rs117031158	G	A	0.012	0.251	0.044	3.70E-09	1.23E-05	5.01
rs2220001	T	G	0.335	-0.055	0.011	1.00E-09	1.22E-05	5.00
rs151059905	A	C	0.026	-0.164	0.033	1.40E-09	1.22E-05	4.98
rs143777421	C	A	0.053	-0.120	0.022	3.20E-09	1.21E-05	4.97
rs72729575	T	C	0.021	-0.186	0.033	1.30E-08	1.21E-05	4.93
rs6460094	A	C	0.409	0.055	0.011	1.60E-09	1.20E-05	4.92
rs11166447	T	C	0.359	0.055	0.011	1.60E-09	1.20E-05	4.91
rs73129515	G	T	0.144	-0.077	0.011	1.70E-09	1.20E-05	4.89
rs35660593	C	CA	0.143	-0.077	0.011	6.10E-09	1.19E-05	4.88
rs146933081	C	A	0.011	0.262	0.044	4.50E-09	1.19E-05	4.87
rs68112593	C	A	0.243	-0.066	0.011	1.70E-09	1.19E-05	4.87
rs7195055	C	T	0.015	0.219	0.033	5.90E-09	1.17E-05	4.80

SNP	Effect allele	Alternative allele	Frequency of effect allele	β-coefficient with HbA1c (mmol/mol)			R2	F-statistics
				β-coefficient	Standard error	P-value		
rs10657263	C	G	0.455	0.055	0.011	2.40E-09	1.17E-05	4.79
rs77020674	C	A	0.028	0.164	0.033	7.70E-09	1.17E-05	4.77
rs879619	G	A	0.136	-0.077	0.011	3.10E-09	1.16E-05	4.76
rs16864320	A	T	0.111	0.087	0.011	2.70E-09	1.16E-05	4.76
rs2966083	G	T	0.452	0.055	0.011	3.10E-09	1.16E-05	4.74
rs11454405	A	AT	0.388	0.055	0.011	5.20E-09	1.16E-05	4.74
rs142391093	A	G	0.245	-0.066	0.011	4.50E-09	1.16E-05	4.73
rs9462407	T	C	0.113	-0.087	0.011	3.10E-09	1.16E-05	4.73
rs148644119	A	ACT	0.079	0.098	0.022	4.50E-09	1.15E-05	4.72
rs2987456	T	C	0.057	-0.109	0.022	3.20E-09	1.15E-05	4.71
rs62019188	G	A	0.420	0.055	0.011	3.70E-09	1.14E-05	4.68
rs112308555	T	A	0.034	-0.142	0.022	2.60E-08	1.14E-05	4.68
rs12669521	A	G	0.340	0.055	0.011	4.50E-09	1.14E-05	4.66
rs140395138	G	T	0.028	-0.153	0.022	6.30E-09	1.14E-05	4.65
rs7071922	C	G	0.265	0.055	0.011	4.50E-09	1.13E-05	4.62
rs10886014	C	T	0.490	0.055	0.011	4.60E-09	1.13E-05	4.62
rs727405	G	A	0.481	0.055	0.011	5.60E-09	1.12E-05	4.57
rs7727635	G	T	0.152	0.077	0.011	6.00E-09	1.11E-05	4.55
rs11878545	G	T	0.150	-0.077	0.011	7.50E-09	1.11E-05	4.54
rs13300763	G	C	0.147	-0.077	0.011	1.00E-08	1.10E-05	4.50
rs1121980	G	A	0.421	-0.055	0.011	7.10E-09	1.10E-05	4.49
rs2973342	A	G	0.251	-0.055	0.011	8.40E-09	1.09E-05	4.47
rs1983128	G	A	0.323	-0.055	0.011	1.70E-08	1.09E-05	4.46
rs12445820	C	G	0.148	0.077	0.011	8.70E-09	1.09E-05	4.45
rs72790097	T	C	0.032	-0.142	0.022	2.20E-08	1.09E-05	4.45
rs231360	C	T	0.392	-0.055	0.011	9.30E-09	1.09E-05	4.45
rs35537689	G	GA	0.307	-0.055	0.011	1.00E-08	1.08E-05	4.42
rs3743380	G	C	0.384	-0.055	0.011	1.20E-08	1.08E-05	4.41

SNP	Effect allele	Alternative allele	Frequency of effect allele	β-coefficient with HbA1c (mmol/mol)			R2	F-statistics
				β-coefficient	Standard error	P-value		
rs7222963	G	C	0.066	0.098	0.022	9.70E-09	1.08E-05	4.41
rs1508615	T	C	0.486	0.055	0.011	1.00E-08	1.08E-05	4.40
rs3784634	C	T	0.437	0.055	0.011	1.00E-08	1.08E-05	4.40
rs76992245	C	G	0.093	0.087	0.011	2.20E-08	1.07E-05	4.37
rs1866476	T	C	0.268	0.055	0.011	1.20E-08	1.07E-05	4.37
rs112484505	G	A	0.020	0.175	0.033	2.80E-08	1.06E-05	4.35
rs35039467	G	C	0.117	0.077	0.011	2.40E-08	1.06E-05	4.33
rs73368485	G	T	0.079	-0.087	0.011	1.30E-08	1.06E-05	4.32
rs10998831	G	A	0.009	-0.262	0.044	2.70E-08	1.05E-05	4.31
rs34592828	G	A	0.045	0.120	0.022	1.70E-08	1.04E-05	4.26
rs6953344	A	G	0.246	-0.055	0.011	1.90E-08	1.04E-05	4.25
rs145016347	T	C	0.031	-0.142	0.022	3.30E-08	1.04E-05	4.24
rs35024453	C	CT	0.139	-0.077	0.011	2.20E-08	1.03E-05	4.23
rs34865291	A	G	0.059	-0.109	0.022	4.20E-08	1.03E-05	4.22
rs10087219	G	A	0.102	-0.077	0.011	2.90E-08	1.02E-05	4.16
rs532854	G	C	0.310	0.055	0.011	2.50E-08	1.02E-05	4.16
rs373130596	CTATT	C	0.039	0.131	0.022	3.70E-08	1.01E-05	4.11
rs7197746	A	T	0.094	0.087	0.011	3.20E-08	1.00E-05	4.11
rs10908278	T	A	0.485	0.044	0.011	3.70E-08	1.00E-05	4.10
rs28483956	G	A	0.045	-0.120	0.022	3.60E-08	9.98E-06	4.08
rs3948593	T	C	0.163	0.066	0.011	4.70E-08	9.97E-06	4.08
rs4279506	G	C	0.374	0.055	0.011	4.70E-08	9.92E-06	4.06
rs17122779	A	G	0.229	-0.055	0.011	4.50E-08	9.78E-06	4.00
Erythrocytic variants:								
rs5030918	A	C	0.026	1.191	0.033	0.00E+00	6.12E-04	250.29
rs150705486	G	A	0.016	1.191	0.033	6.20E-231	3.67E-04	150.26
rs2748424	C	G	0.189	-0.372	0.011	6.20E-232	3.53E-04	144.34
rs113373052	C	T	0.313	-0.306	0.011	9.00E-229	3.42E-04	140.07

SNP	Effect allele	Alternative allele	Frequency of effect allele	β-coefficient with HbA1c (mmol/mol)			R2	F-statistics
				β-coefficient	Standard error	P-value		
rs79220007	T	C	0.077	0.536	0.022	2.80E-227	3.40E-04	139.05
rs857725	T	G	0.266	-0.317	0.011	5.70E-222	3.31E-04	135.30
rs4737010	G	A	0.228	-0.328	0.011	2.10E-217	3.26E-04	133.36
rs200589393	CT	C	0.421	0.273	0.011	9.30E-201	3.08E-04	126.10
rs855791	A	G	0.439	0.262	0.011	1.40E-191	2.87E-04	117.60
rs12819124	C	A	0.473	0.251	0.011	2.20E-174	2.61E-04	106.77
rs551118	C	G	0.422	-0.240	0.011	6.70E-150	2.30E-04	94.17
rs198851	T	G	0.151	-0.328	0.011	8.80E-154	2.29E-04	93.53
rs34265667	G	A	0.035	0.623	0.022	6.40E-146	2.17E-04	88.89
rs9299503	G	A	0.488	-0.230	0.011	2.10E-125	2.14E-04	87.43
rs1849134	A	T	0.305	0.230	0.011	2.20E-120	1.79E-04	73.14
rs61750929	C	T	0.055	0.415	0.022	2.00E-101	1.50E-04	61.52
rs35365035	T	C	0.392	-0.186	0.011	1.20E-95	1.41E-04	57.64
rs13266634	C	T	0.313	0.197	0.011	2.10E-91	1.35E-04	55.10
rs12876143	T	C	0.089	-0.317	0.011	1.30E-90	1.34E-04	54.68
rs76895963	T	G	0.021	0.612	0.033	1.20E-73	1.31E-04	53.55
rs11735662	C	T	0.034	0.459	0.022	9.50E-80	1.17E-04	47.81
rs635634	C	T	0.185	-0.208	0.011	2.30E-72	1.07E-04	43.73
rs453922	T	C	0.275	-0.175	0.011	1.60E-68	1.06E-04	43.42
rs11438979	A	AG	0.419	0.164	0.011	1.10E-70	1.04E-04	42.54
rs10823340	C	T	0.249	-0.175	0.011	7.60E-67	9.79E-05	40.06
rs371973771	G	A	0.130	-0.230	0.011	2.70E-65	9.75E-05	39.86
rs9389268	A	G	0.260	0.175	0.011	6.00E-63	9.25E-05	37.85
rs11530658	A	G	0.100	0.240	0.011	2.30E-60	8.84E-05	36.17
rs6602913	A	C	0.367	-0.142	0.011	1.10E-56	8.29E-05	33.90
rs1175550	A	G	0.231	0.164	0.011	1.30E-52	7.86E-05	32.13
rs112694524	G	A	0.073	0.251	0.022	1.00E-48	7.30E-05	29.86
rs56267269	C	T	0.411	0.131	0.011	2.90E-50	7.28E-05	29.78

SNP	Effect allele	Alternative allele	Frequency of effect allele	β-coefficient with HbA1c (mmol/mol)			R2	F-statistics
				β-coefficient	Standard error	P-value		
rs78565860	T	C	0.047	0.306	0.022	1.70E-49	7.17E-05	29.32
rs9269095	G	A	0.301	-0.142	0.011	3.80E-43	7.12E-05	29.12
rs7137828	C	T	0.482	-0.131	0.011	1.00E-48	7.08E-05	28.97
rs112100783	G	A	0.034	0.350	0.022	3.00E-45	6.82E-05	27.88
rs34552339	A	AG	0.351	0.131	0.011	7.80E-47	6.77E-05	27.69
rs7224870	A	G	0.203	0.153	0.011	3.50E-46	6.65E-05	27.21
rs369333540	C	CGGGA	0.174	0.164	0.011	3.90E-40	6.41E-05	26.21
rs4758633	A	G	0.456	-0.120	0.011	3.90E-44	6.38E-05	26.10
rs1642762	C	T	0.437	0.120	0.011	6.20E-43	6.27E-05	25.66
rs7451008	T	C	0.261	-0.142	0.011	2.30E-42	6.13E-05	25.09
rs144658542	A	AT	0.199	-0.153	0.011	6.50E-41	5.86E-05	23.97
rs28505677	C	G	0.237	0.142	0.011	3.00E-39	5.71E-05	23.36
rs6014993	A	G	0.487	-0.120	0.011	1.90E-39	5.69E-05	23.29
rs12667932	G	A	0.080	0.219	0.011	3.10E-39	5.64E-05	23.06
rs340882	C	G	0.379	-0.120	0.011	2.30E-38	5.55E-05	22.71
rs75372982	A	G	0.287	0.131	0.011	3.00E-37	5.47E-05	22.36
rs6808166	G	A	0.426	-0.109	0.011	4.50E-37	5.34E-05	21.84
rs11655029	T	C	0.305	0.120	0.011	5.20E-37	5.33E-05	21.82
rs1967604	A	G	0.287	0.120	0.011	7.10E-37	5.31E-05	21.70
rs12771121	C	T	0.207	-0.142	0.011	1.60E-35	5.19E-05	21.24
rs429961	T	A	0.184	0.142	0.011	4.30E-35	5.17E-05	21.14
rs115340137	T	C	0.009	0.590	0.044	5.40E-36	5.14E-05	21.03
rs2143918	A	C	0.471	0.109	0.011	7.30E-36	5.14E-05	21.00
rs9872660	G	T	0.166	-0.153	0.011	4.70E-35	5.12E-05	20.93
rs9258357	T	C	0.164	-0.142	0.011	1.40E-34	4.92E-05	20.14
rs3132513	A	G	0.439	-0.109	0.011	7.70E-34	4.80E-05	19.64
rs34417262	G	A	0.191	-0.131	0.011	1.40E-33	4.80E-05	19.61
rs78588343	G	A	0.180	0.142	0.011	9.50E-33	4.66E-05	19.07

SNP	Effect allele	Alternative allele	Frequency of effect allele	β-coefficient with HbA1c (mmol/mol)			R2	F-statistics
				β-coefficient	Standard error	P-value		
rs112802399	G	T	0.033	0.295	0.022	2.10E-32	4.60E-05	18.81
rs12341913	A	T	0.122	-0.153	0.011	2.80E-22	4.45E-05	18.22
rs174557	A	G	0.310	0.109	0.011	4.50E-31	4.39E-05	17.97
rs73396237	C	G	0.148	0.142	0.011	1.20E-30	4.33E-05	17.72
rs10840425	A	C	0.415	0.098	0.011	1.50E-30	4.33E-05	17.71
rs12941965	A	C	0.223	0.120	0.011	3.70E-30	4.28E-05	17.52
rs2285005	G	A	0.340	-0.109	0.011	1.20E-29	4.19E-05	17.14
rs112601576	C	T	0.248	-0.120	0.011	1.30E-28	4.16E-05	17.01
rs3811444	C	T	0.333	-0.109	0.011	3.50E-29	4.11E-05	16.80
rs8050500	T	C	0.446	0.098	0.011	1.40E-28	4.03E-05	16.47
rs247826	C	T	0.223	-0.120	0.011	5.90E-28	3.97E-05	16.22
rs13215994	C	T	0.281	0.109	0.011	2.10E-27	3.86E-05	15.79
rs1894474	A	G	0.481	0.098	0.011	7.20E-27	3.80E-05	15.53
rs59168150	C	T	0.288	-0.109	0.011	1.30E-16	3.79E-05	15.51
rs2375279	T	C	0.151	0.131	0.011	8.70E-27	3.77E-05	15.42
rs12921546	G	A	0.282	-0.109	0.011	1.30E-26	3.74E-05	15.31
rs2858010	T	C	0.340	0.098	0.011	2.10E-26	3.73E-05	15.25
rs60757417	C	G	0.060	-0.197	0.022	6.70E-26	3.70E-05	15.14
rs28856692	G	A	0.444	-0.098	0.011	8.60E-16	3.69E-05	15.10
rs79755767	G	A	0.100	0.153	0.011	8.30E-26	3.68E-05	15.03
rs267738	T	G	0.221	0.109	0.011	1.60E-25	3.57E-05	14.62
rs2954021	A	G	0.494	-0.087	0.011	2.30E-25	3.55E-05	14.53
rs72811487	G	A	0.035	0.251	0.022	3.00E-25	3.52E-05	14.41
rs10901252	G	C	0.060	0.197	0.022	5.90E-25	3.51E-05	14.34
rs2060779	G	A	0.222	0.109	0.011	5.80E-25	3.50E-05	14.33
rs72939920	A	T	0.235	0.109	0.011	1.90E-24	3.43E-05	14.04
rs7732130	G	A	0.319	0.098	0.011	2.50E-24	3.40E-05	13.90
rs155766	C	T	0.366	0.098	0.011	3.00E-18	3.37E-05	13.77

SNP	Effect allele	Alternative allele	Frequency of effect allele	β-coefficient with HbA1c (mmol/mol)			R2	F-statistics
				β-coefficient	Standard error	P-value		
rs79046494	G	A	0.343	-0.098	0.011	7.00E-24	3.33E-05	13.62
rs9994509	A	G	0.392	0.087	0.011	1.70E-23	3.29E-05	13.44
rs11235688	G	A	0.416	0.087	0.011	1.80E-23	3.28E-05	13.42
rs4791641	C	T	0.495	0.087	0.011	2.00E-23	3.26E-05	13.33
rs59153558	A	G	0.280	-0.098	0.011	4.50E-23	3.22E-05	13.15
rs10501320	G	C	0.265	0.098	0.011	4.20E-23	3.21E-05	13.14
rs12607898	A	G	0.254	-0.098	0.011	5.30E-23	3.21E-05	13.11
rs13434265	C	T	0.416	0.087	0.011	8.60E-23	3.19E-05	13.04
rs117370443	T	C	0.039	-0.219	0.022	1.00E-22	3.17E-05	12.96
rs6055955	C	T	0.493	0.087	0.011	1.10E-22	3.16E-05	12.90
rs13167071	G	C	0.360	-0.087	0.011	1.30E-22	3.15E-05	12.86
rs1260334	C	A	0.434	-0.087	0.011	1.40E-22	3.13E-05	12.79
rs142621189	C	T	0.010	-0.437	0.044	3.50E-21	3.06E-05	12.51
rs11643024	A	G	0.308	0.087	0.011	1.40E-21	2.99E-05	12.22
rs1047912	C	T	0.302	-0.087	0.011	1.40E-21	2.98E-05	12.17
rs8110787	C	T	0.392	0.087	0.011	1.40E-21	2.97E-05	12.14
rs41276588	G	A	0.286	-0.087	0.011	4.20E-21	2.95E-05	12.07
rs7572278	T	A	0.204	-0.098	0.011	5.80E-21	2.90E-05	11.84
rs35188965	C	T	0.418	-0.087	0.011	1.70E-20	2.82E-05	11.53
rs71326967	A	G	0.344	-0.087	0.011	2.90E-20	2.82E-05	11.51
rs201675920	T	TG	0.243	-0.098	0.011	8.60E-20	2.78E-05	11.37
rs34878143	T	C	0.024	0.262	0.033	5.10E-20	2.78E-05	11.36
rs459547	G	A	0.291	-0.087	0.011	4.00E-20	2.78E-05	11.36
rs1126930	G	C	0.036	0.219	0.022	3.80E-20	2.77E-05	11.33
rs9270461	C	T	0.365	-0.087	0.011	7.90E-18	2.76E-05	11.30
rs6773529	T	C	0.169	0.109	0.011	6.00E-20	2.76E-05	11.29
rs6736362	C	T	0.437	-0.077	0.011	5.70E-20	2.75E-05	11.26
rs7036656	C	T	0.278	-0.087	0.011	6.10E-20	2.74E-05	11.22

SNP	Effect allele	Alternative allele	Frequency of effect allele	β-coefficient with HbA1c (mmol/mol)			R2	F-statistics
				β-coefficient	Standard error	P-value		
rs7820334	C	T	0.311	0.087	0.011	1.30E-19	2.72E-05	11.12
rs2572207	C	T	0.239	-0.098	0.011	8.10E-20	2.72E-05	11.11
rs17803780	T	C	0.212	0.098	0.011	4.30E-19	2.62E-05	10.71
rs3744222	C	T	0.245	0.087	0.011	3.70E-18	2.61E-05	10.68
rs5995382	C	T	0.483	-0.077	0.011	1.10E-18	2.59E-05	10.58
rs183034862	C	T	0.025	-0.251	0.033	3.30E-17	2.51E-05	10.28
rs1569780	A	C	0.395	0.077	0.011	4.10E-18	2.50E-05	10.24
rs199737194	G	A	0.040	-0.197	0.033	4.40E-11	2.49E-05	10.18
rs7606173	G	C	0.432	0.077	0.011	3.50E-18	2.47E-05	10.12
rs4660342	T	G	0.423	-0.077	0.011	1.20E-15	2.44E-05	9.98
rs11545157	T	C	0.114	0.120	0.011	7.60E-18	2.43E-05	9.93
rs941718	T	C	0.277	-0.087	0.011	3.10E-17	2.34E-05	9.55
rs74685815	C	T	0.028	0.230	0.033	2.50E-16	2.32E-05	9.48
rs6785881	C	T	0.482	0.077	0.011	4.80E-17	2.31E-05	9.47
rs634454	T	G	0.211	-0.087	0.011	7.10E-16	2.28E-05	9.32
rs246232	C	G	0.315	-0.077	0.011	1.00E-16	2.28E-05	9.31
rs998584	C	A	0.481	-0.077	0.011	1.10E-16	2.26E-05	9.26
rs11614506	T	C	0.229	0.087	0.011	8.80E-17	2.26E-05	9.26
rs2270927	C	G	0.094	-0.131	0.011	9.00E-17	2.26E-05	9.25
rs592423	A	C	0.446	0.077	0.011	1.20E-16	2.25E-05	9.19
rs584007	A	G	0.357	0.077	0.011	2.10E-16	2.23E-05	9.10
rs7047279	T	C	0.432	0.077	0.011	3.00E-16	2.21E-05	9.04
rs7546390	T	C	0.095	-0.120	0.011	9.50E-16	2.17E-05	8.88
rs11043299	T	C	0.376	-0.077	0.011	5.60E-14	2.14E-05	8.75
rs11851199	A	G	0.073	0.142	0.022	1.00E-15	2.14E-05	8.74
rs2041601	G	C	0.091	-0.120	0.011	8.60E-16	2.13E-05	8.70
rs192095068	G	A	0.171	0.098	0.011	4.20E-15	2.09E-05	8.54
rs181124493	C	T	0.022	-0.240	0.033	3.20E-15	2.09E-05	8.54

SNP	Effect allele	Alternative allele	Frequency of effect allele	β-coefficient with HbA1c (mmol/mol)			R2	F-statistics
				β-coefficient	Standard error	P-value		
rs2060985	A	G	0.463	0.066	0.011	1.40E-15	2.09E-05	8.54
rs7720275	T	C	0.170	-0.098	0.011	1.90E-15	2.07E-05	8.47
rs7747023	A	G	0.159	-0.098	0.011	2.30E-15	2.05E-05	8.40
rs2005079	G	A	0.335	-0.077	0.011	3.50E-15	2.03E-05	8.31
rs117826678	A	G	0.037	0.186	0.022	1.30E-13	2.03E-05	8.30
rs7309882	G	T	0.370	0.077	0.011	1.20E-14	2.02E-05	8.26
rs3780962	A	G	0.467	-0.066	0.011	5.80E-15	2.01E-05	8.22
rs13063578	T	A	0.399	-0.066	0.011	5.20E-15	2.01E-05	8.21
rs115806594	C	A	0.016	0.273	0.033	6.10E-14	2.00E-05	8.17
rs9324022	T	C	0.209	-0.087	0.011	3.90E-14	1.96E-05	8.04
rs34221578	C	T	0.077	-0.131	0.022	2.40E-14	1.94E-05	7.94
rs9389212	A	G	0.209	-0.087	0.011	1.50E-14	1.94E-05	7.93
rs4665710	A	C	0.206	0.087	0.011	1.50E-14	1.94E-05	7.92
rs9264277	T	C	0.368	-0.066	0.011	1.90E-14	1.94E-05	7.92
rs117305036	G	A	0.056	0.153	0.022	2.80E-13	1.92E-05	7.86
rs6937957	T	C	0.195	-0.087	0.011	2.40E-14	1.91E-05	7.79
rs111631066	G	A	0.016	0.273	0.033	1.60E-13	1.90E-05	7.79
rs11658587	C	T	0.037	0.175	0.022	4.00E-14	1.87E-05	7.63
rs35979828	C	T	0.069	0.131	0.022	4.80E-14	1.86E-05	7.59
rs12743834	T	C	0.397	-0.066	0.011	6.70E-14	1.84E-05	7.52
rs12531645	G	A	0.284	-0.077	0.011	6.70E-14	1.84E-05	7.52
rs1570360	A	G	0.329	-0.066	0.011	7.40E-14	1.83E-05	7.50
rs79985479	C	A	0.173	0.087	0.011	5.00E-12	1.80E-05	7.37
rs2737263	G	T	0.285	0.077	0.011	2.10E-13	1.77E-05	7.24
rs17531978	T	C	0.129	-0.098	0.011	2.30E-13	1.76E-05	7.21
rs4480845	T	C	0.359	0.066	0.011	3.30E-13	1.76E-05	7.20
rs1416218	C	G	0.472	-0.066	0.011	3.30E-13	1.75E-05	7.15
rs117351529	T	C	0.082	-0.120	0.011	3.70E-13	1.75E-05	7.14

SNP	Effect allele	Alternative allele	Frequency of effect allele	β-coefficient with HbA1c (mmol/mol)			R2	F-statistics
				β-coefficient	Standard error	P-value		
rs374841001	T	TA	0.316	-0.066	0.011	4.60E-13	1.75E-05	7.14
rs60968356	T	C	0.049	-0.153	0.022	2.80E-12	1.73E-05	7.08
rs10421794	T	C	0.402	-0.066	0.011	3.90E-13	1.73E-05	7.06
rs11237477	A	T	0.158	0.087	0.011	4.30E-13	1.72E-05	7.02
rs12925612	G	C	0.012	-0.295	0.044	2.80E-12	1.71E-05	6.98
rs4411786	T	C	0.262	0.077	0.011	7.30E-13	1.69E-05	6.92
rs17462893	A	G	0.101	-0.109	0.011	7.80E-13	1.68E-05	6.89
rs701848	T	C	0.390	0.066	0.011	1.10E-12	1.67E-05	6.83
rs12096473	C	T	0.070	0.120	0.022	1.80E-12	1.64E-05	6.72
rs10752943	C	T	0.432	0.066	0.011	1.90E-12	1.63E-05	6.66
rs56271783	G	C	0.045	-0.153	0.022	3.20E-12	1.62E-05	6.63
rs116248380	C	T	0.021	-0.219	0.033	2.30E-12	1.61E-05	6.58
rs12525479	G	A	0.063	-0.131	0.022	3.10E-12	1.60E-05	6.56
rs11844552	A	G	0.024	0.208	0.033	3.60E-12	1.60E-05	6.55
rs141820703	T	C	0.017	-0.240	0.033	1.20E-11	1.60E-05	6.54
rs9824251	C	T	0.136	-0.087	0.011	3.40E-12	1.59E-05	6.52
rs9468693	T	C	0.071	-0.120	0.022	3.40E-12	1.59E-05	6.49
rs76498834	C	T	0.045	-0.153	0.022	4.10E-12	1.58E-05	6.45
rs73564493	G	A	0.072	0.120	0.022	4.40E-12	1.57E-05	6.43
rs117311034	G	T	0.096	-0.098	0.011	7.20E-12	1.56E-05	6.39
rs150848796	G	T	0.022	0.208	0.033	2.00E-11	1.55E-05	6.35
rs351978	A	G	0.419	0.066	0.011	1.20E-11	1.55E-05	6.35
rs6598047	T	C	0.231	0.077	0.011	3.80E-09	1.55E-05	6.33
rs12878001	T	G	0.160	-0.087	0.011	9.10E-12	1.53E-05	6.26
rs11557154	C	T	0.127	0.087	0.011	1.00E-11	1.52E-05	6.20
rs73210702	G	A	0.036	-0.164	0.022	2.10E-11	1.50E-05	6.14
rs10923372	T	C	0.246	0.066	0.011	1.50E-11	1.50E-05	6.13
rs6719549	G	A	0.192	0.077	0.011	2.40E-11	1.49E-05	6.10

SNP	Effect allele	Alternative allele	Frequency of effect allele	β-coefficient with HbA1c (mmol/mol)			R2	F-statistics
				β-coefficient	Standard error	P-value		
rs2608073	C	T	0.084	0.109	0.022	7.90E-11	1.49E-05	6.08
rs36110254	C	CA	0.283	-0.066	0.011	1.60E-11	1.49E-05	6.08
rs72688461	G	T	0.326	0.066	0.011	4.60E-11	1.48E-05	6.04
rs299646	G	C	0.422	0.055	0.011	2.00E-11	1.47E-05	6.02
rs36084354	G	A	0.093	0.098	0.011	2.10E-11	1.47E-05	6.01
rs2879904	C	T	0.122	0.087	0.011	4.00E-10	1.46E-05	5.96
rs75475570	G	A	0.182	0.077	0.011	2.90E-11	1.46E-05	5.95
rs1354034	T	C	0.399	-0.066	0.011	2.70E-11	1.45E-05	5.93
rs113349196	A	G	0.107	-0.098	0.011	5.40E-11	1.44E-05	5.90
rs1399627	A	G	0.348	-0.066	0.011	3.70E-11	1.44E-05	5.88
rs12107581	C	T	0.344	0.066	0.011	4.50E-11	1.42E-05	5.82
rs34302523	C	A	0.434	-0.055	0.011	4.50E-11	1.42E-05	5.82
rs35133002	A	T	0.286	0.066	0.011	4.60E-11	1.42E-05	5.82
rs4812826	G	A	0.306	-0.066	0.011	5.00E-11	1.42E-05	5.81
rs61047863	G	A	0.219	0.066	0.011	7.60E-11	1.42E-05	5.81
rs45607834	C	T	0.103	-0.098	0.011	5.30E-11	1.42E-05	5.80
rs151059129	C	CA	0.349	-0.066	0.011	5.70E-11	1.42E-05	5.79
rs711244	C	T	0.420	0.055	0.011	4.80E-11	1.41E-05	5.79
rs2026882	C	T	0.162	0.077	0.011	5.50E-11	1.41E-05	5.78
rs2394322	C	T	0.336	-0.066	0.011	1.80E-10	1.38E-05	5.65
rs6503492	T	A	0.125	0.087	0.011	1.30E-10	1.37E-05	5.61
rs2641436	C	T	0.290	0.066	0.011	1.00E-10	1.37E-05	5.61
rs12133641	A	G	0.410	0.055	0.011	9.40E-11	1.37E-05	5.61
rs71420477	G	C	0.019	-0.208	0.033	4.40E-10	1.37E-05	5.61
rs7099229	G	A	0.266	-0.066	0.011	1.10E-10	1.37E-05	5.59
rs10222374	C	T	0.217	-0.066	0.011	1.70E-10	1.37E-05	5.59
rs34165383	C	G	0.096	0.098	0.011	1.40E-10	1.37E-05	5.58
rs143509164	C	CATT	0.290	-0.066	0.011	3.30E-10	1.36E-05	5.58

SNP	Effect allele	Alternativ e allele	Frequen cy of effect allele	β-coefficient with HbA1c (mmol/mol)			R2	F-statistics
				β-coefficient	Standar d error	P-value		
rs11294964 4	T	C	0.150	-0.077	0.011	1.30E-10	1.36E-05	5.57
rs9787306	C	T	0.211	0.066	0.011	1.30E-10	1.36E-05	5.55
rs10092989	G	T	0.164	-0.077	0.011	2.20E-10	1.36E-05	5.55
rs9900933	T	C	0.343	0.055	0.011	1.40E-10	1.36E-05	5.55
rs1476171	T	C	0.234	-0.066	0.011	1.40E-10	1.35E-05	5.54
rs755249	C	T	0.233	-0.066	0.011	1.40E-10	1.35E-05	5.52
rs8176059	G	A	0.011	0.273	0.044	1.40E-10	1.35E-05	5.52
rs75866363	G	A	0.072	0.109	0.022	1.50E-10	1.35E-05	5.50
rs551720	A	T	0.367	-0.055	0.011	2.00E-10	1.34E-05	5.49
rs1891058	G	A	0.394	-0.055	0.011	2.10E-10	1.33E-05	5.45
rs12992076	T	C	0.102	0.098	0.011	2.90E-10	1.33E-05	5.45
rs909134	T	C	0.316	0.066	0.011	2.00E-10	1.32E-05	5.41
rs9423289	C	T	0.423	-0.055	0.011	2.20E-10	1.32E-05	5.40
rs14742804 0	G	A	0.043	-0.142	0.022	1.60E-09	1.32E-05	5.39
rs6922503	C	T	0.277	0.066	0.011	2.40E-10	1.32E-05	5.39
rs836468	T	A	0.310	0.066	0.011	2.20E-10	1.32E-05	5.39
rs2294856	G	C	0.355	-0.055	0.011	2.10E-10	1.32E-05	5.38
rs11707190 5	G	A	0.010	-0.273	0.044	5.10E-10	1.31E-05	5.37
rs6087995	A	C	0.452	-0.055	0.011	3.30E-10	1.30E-05	5.32
rs157227	A	G	0.370	0.055	0.011	3.10E-10	1.30E-05	5.32
rs2867932	A	G	0.398	-0.055	0.011	3.40E-10	1.29E-05	5.29
rs706848	G	A	0.439	-0.055	0.011	3.80E-10	1.29E-05	5.26
rs6073967	C	T	0.332	0.055	0.011	4.30E-10	1.28E-05	5.22
rs72785418	G	C	0.081	0.098	0.022	6.60E-10	1.28E-05	5.22
rs9314323	A	G	0.300	-0.066	0.011	5.20E-10	1.28E-05	5.22
rs55938136	A	G	0.226	0.066	0.011	4.20E-10	1.28E-05	5.22
rs12943904	G	C	0.306	0.055	0.011	5.60E-10	1.27E-05	5.18
rs1030097	C	G	0.495	-0.055	0.011	7.30E-10	1.26E-05	5.16

SNP	Effect allele	Alternative allele	Frequency of effect allele	β-coefficient with HbA1c (mmol/mol)			R2	F-statistics
				β-coefficient	Standard error	P-value		
rs9701457	C	T	0.364	0.055	0.011	6.30E-10	1.26E-05	5.16
rs56395926	G	A	0.229	0.066	0.011	6.70E-10	1.26E-05	5.14
rs2304234	T	C	0.424	0.055	0.011	7.30E-10	1.25E-05	5.13
rs6063050	T	C	0.284	0.066	0.011	7.50E-10	1.25E-05	5.12
rs488270	C	T	0.352	-0.055	0.011	6.70E-10	1.25E-05	5.11
rs12600879	A	G	0.382	0.055	0.011	1.30E-09	1.25E-05	5.11
rs11620577	C	T	0.243	-0.066	0.011	7.30E-10	1.24E-05	5.08
rs7220535	G	A	0.388	0.055	0.011	9.10E-10	1.23E-05	5.04
rs62427625	C	T	0.136	0.077	0.011	1.60E-09	1.22E-05	4.97
rs690604	G	T	0.081	0.098	0.022	4.90E-09	1.22E-05	4.97
rs149142833	C	T	0.156	-0.077	0.011	1.80E-09	1.21E-05	4.94
rs75423791	T	A	0.055	0.120	0.022	1.70E-09	1.20E-05	4.92
rs9477866	A	G	0.409	0.055	0.011	2.30E-09	1.20E-05	4.91
rs2934701	A	T	0.427	0.055	0.011	1.60E-09	1.20E-05	4.90
rs73625323	A	G	0.136	-0.077	0.011	3.20E-09	1.20E-05	4.90
rs1049654	A	C	0.438	0.055	0.011	1.70E-09	1.20E-05	4.89
rs8045833	G	A	0.269	-0.055	0.011	1.80E-09	1.19E-05	4.86
rs6844176	T	C	0.456	-0.055	0.011	2.30E-09	1.18E-05	4.85
rs62136856	A	G	0.284	-0.055	0.011	2.50E-09	1.16E-05	4.76
rs2736182	G	A	0.018	-0.197	0.033	2.40E-09	1.16E-05	4.76
rs3790455	C	T	0.344	0.055	0.011	2.70E-09	1.16E-05	4.75
rs13022216	G	A	0.475	-0.055	0.011	3.70E-09	1.16E-05	4.73
rs116656374	G	T	0.038	-0.142	0.022	2.90E-09	1.15E-05	4.72
rs2811434	T	G	0.214	0.066	0.011	4.10E-09	1.15E-05	4.68
rs318468	T	C	0.381	-0.055	0.011	4.00E-09	1.14E-05	4.65
rs75417300	A	C	0.185	-0.066	0.011	1.40E-08	1.13E-05	4.62
rs111970489	GA	G	0.193	-0.066	0.011	6.70E-09	1.12E-05	4.59
rs1427297	C	T	0.319	0.055	0.011	6.00E-09	1.11E-05	4.53

SNP	Effect allele	Alternative allele	Frequency of effect allele	β-coefficient with HbA1c (mmol/mol)			R2	F-statistics
				β-coefficient	Standard error	P-value		
rs1047891	C	A	0.316	0.055	0.011	6.10E-09	1.11E-05	4.52
rs1412837	G	A	0.242	-0.055	0.011	1.20E-08	1.10E-05	4.50
rs116986845	C	T	0.023	-0.175	0.033	6.90E-09	1.10E-05	4.49
rs367070	A	G	0.228	-0.066	0.011	8.50E-09	1.10E-05	4.48
rs148912436	T	C	0.012	0.240	0.044	7.80E-09	1.09E-05	4.47
rs2503699	T	C	0.318	0.055	0.011	7.60E-09	1.09E-05	4.47
rs4135183	C	T	0.145	0.077	0.011	1.10E-08	1.08E-05	4.41
rs34952318	G	A	0.049	0.120	0.022	1.60E-08	1.07E-05	4.39
rs73114873	T	C	0.120	-0.077	0.011	1.00E-08	1.07E-05	4.38
rs927984	T	C	0.153	0.066	0.011	1.10E-08	1.07E-05	4.36
rs7787060	T	C	0.307	-0.055	0.011	1.90E-08	1.06E-05	4.35
rs1490384	C	T	0.497	-0.055	0.011	1.40E-08	1.06E-05	4.32
rs12522289	A	G	0.081	-0.087	0.022	2.10E-08	1.06E-05	4.32
rs2771425	A	G	0.384	0.055	0.011	1.70E-08	1.05E-05	4.29
rs6054427	G	A	0.379	0.055	0.011	1.80E-08	1.04E-05	4.27
rs75503064	G	A	0.079	0.087	0.022	2.10E-08	1.04E-05	4.26
rs13007705	C	T	0.443	0.055	0.011	2.10E-08	1.04E-05	4.24
rs145818374	C	T	0.024	0.164	0.033	3.30E-08	1.03E-05	4.20
rs941405	C	G	0.363	-0.055	0.011	2.50E-08	1.02E-05	4.19
rs77542162	A	G	0.023	0.164	0.033	2.30E-08	1.02E-05	4.19
rs6452790	G	A	0.227	0.055	0.011	2.60E-08	1.02E-05	4.19
rs3742321	T	C	0.226	0.055	0.011	2.50E-08	1.02E-05	4.18
rs1968956	G	T	0.041	0.120	0.022	2.30E-08	1.02E-05	4.18
rs4759319	G	T	0.368	0.055	0.011	2.50E-08	1.02E-05	4.17
rs11387142	C	CT	0.216	0.055	0.011	3.50E-08	1.02E-05	4.16
rs9836493	T	C	0.351	-0.055	0.011	2.60E-08	1.02E-05	4.16
rs17138703	A	G	0.213	0.055	0.011	2.80E-08	1.01E-05	4.13
rs72801470	G	C	0.256	0.055	0.011	3.20E-08	1.01E-05	4.12

SNP	Effect allele	Alternative allele	Frequency of effect allele	β-coefficient with HbA1c (mmol/mol)			R2	F-statistics
				β-coefficient	Standard error	P-value		
rs4654386	T	C	0.199	-0.066	0.011	3.30E-08	1.00E-05	4.10
rs6084653	C	G	0.391	0.055	0.011	3.70E-08	1.00E-05	4.09
rs139727420	C	CAT	0.378	-0.055	0.011	3.20E-08	1.00E-05	4.09
rs7952868	A	G	0.246	-0.055	0.011	3.90E-08	9.91E-06	4.05
rs17305657	T	C	0.103	0.077	0.011	3.80E-08	9.90E-06	4.05
rs58950470	G	T	0.352	-0.055	0.011	4.10E-08	9.86E-06	4.03
rs11616188	G	A	0.426	0.044	0.011	4.30E-08	9.84E-06	4.02
rs12208868	C	T	0.048	-0.109	0.022	4.90E-08	9.84E-06	4.02
rs2959356	A	G	0.298	-0.055	0.011	4.50E-08	9.81E-06	4.01
rs4809330	A	G	0.334	-0.055	0.011	4.80E-08	9.72E-06	3.98
